# Pilotierung einer elektronischen Todesbescheinigung (eTB-App) – Nutzungserfahrungen von Ärztinnen und Ärzten

**DOI:** 10.1007/s00103-025-04082-w

**Published:** 2025-06-02

**Authors:** Sebastian Brunner, Michaela Hesse, Diana Borucki, Ulrich Vogel, Martin Mücke, Gülay Ateş

**Affiliations:** 1 Institut für Digitale Allgemeinmedizin, Zentrum für Seltene Erkrankungen Aachen (ZSEA), Medizinische Fakultät, RWTH Aachen University, Anstalt des öffentlichen Rechts (AöR), Pauwelsstraße 30, 52074 Aachen, Deutschland; 2 Abteilung K – Kodiersysteme, Bundesinstitut für Arzneimittel und Medizinprodukte (BfArM), Bonn, Deutschland

**Keywords:** Todesursachenstatistik, Bedienungsfreundlichkeit, Mixed-Methods-Studie, Digitale Anwendung, Digitalisierung, Mortality data, Usability, Mixed-methods design, Digital application, Digitalisation

## Abstract

**Hintergrund:**

Todesbescheinigungen werden händisch ausgefüllt und durchlaufen zur Transkription und Kodierung verschiedene Ämter, bevor Todesursachenstatistiken publiziert werden. Die Bescheinigungen sind häufig fehlerhaft und schwer lesbar. In Deutschland existieren 16 verschiedene Formulare je nach Bundesland und alle entsprechen nicht den Vorgaben der Weltgesundheitsorganisation (WHO) zur internationalen Vergleichbarkeit. Gefördert vom Bundesministerium für Gesundheit wurde vom Bundesinstitut für Arzneimittel und Medizinprodukte in Zusammenarbeit mit dem Statistischen Bundesamt eine elektronische Todesbescheinigung als App entwickelt und in einer Testphase in 2 Bundesländern auf Bedienungsfreundlichkeit und Akzeptanz untersucht.

**Methode:**

Es wurde ein Mixed-Methods-Ansatz mit validierten Fragebögen und halbstrukturierten Interviews eingesetzt (02.–09.2023). Die quantitativen Daten wurden deskriptiv mit SPSS und die qualitativen Daten inhaltsanalytisch mit MAXQDA ausgewertet.

**Ergebnisse:**

In 2 Bundesländern füllten 89 von 201 Ärztinnen und Ärzten die Online-Befragung aus und zusätzlich wurden 11 Personen interviewt. Fast alle Befragten (*n* = 70) stehen der Digitalisierung im Gesundheitswesen positiv gegenüber (94 %), sehen einen Nutzen in Abläufen und Organisation (93 %) und haben keine Bedenken hinsichtlich der Datensicherheit (73 %). Die Mehrheit bestätigt die Bedienungsfreundlichkeit der eTB-App. Die qualitativen Interviews bestätigen, dass Prüfung auf Plausibilität, Vermeidung von Fehlern und bessere Lesbarkeit überzeugende Argumente für die App sind.

**Fazit:**

Die eTB-App ist leicht zu bedienen und nützlich. Insbesondere die schnelle Datenübernahme macht die eTB zu einem wichtigen Meilenstein in der Digitalisierung. Für den Einsatz in der Praxis sind Verbesserungen erforderlich.

## Abstract (en)

**Background:**

Funded by the Federal Ministry of Health, the Federal Institute for Drugs and Medical Devices, in cooperation with the Federal Statistical Office, developed an electronic death certificate app and tested it in two federal states to assess its user-friendliness and acceptance.

Death certificates are filled out by hand and pass through various offices for transcription and coding before cause of death statistics are published. The certificates are often incorrect and difficult to read. In Germany, there are 16 different forms depending on the federal state, and none of them meet World Health Organization (WHO) requirements for international comparability.

**Methods:**

A mixed-methods approach was used with validated questionnaires and semi-structured guided interviews (February to September 2023). The quantitative data were analysed descriptively with SPSS and the qualitative data were analysed using content analysis with MAXQDA.

**Results:**

In two federal states, a total of 89 out of 201 physicians completed the surveys and a further 11 were interviewed. Almost all respondents (*n* = 70) have a positive attitude towards digitalisation in healthcare (94%), see a benefit in processes and organisation (93%) and have no concerns about data security (73%). The majority confirmed the user-friendliness of the eTB app. The qualitative data highlighted that plausibility checking, error prevention and improved readability were convincing arguments in favour of the eTB app.

**Conclusion:**

The eTB app is easy to use and useful. In particular, the fast data transfer makes the eTB app an important milestone in digitalization. Improvements are required for use in practice.

## Hintergrund

In Deutschland erfolgt die Erstellung der (inter)nationalen Todesursachenstatistik aktuell auf Basis von 16 unterschiedlichen Formularen, die nicht den von der Weltgesundheitsorganisation (WHO) festgelegten Standards entsprechen. Dies erschwert nationale und internationale Vergleiche und macht eine Anpassung notwendig. Zudem ist anzumerken, dass die Todesbescheinigungen derzeit manuell ausgefüllt werden, was eine postalische Weiterleitung an verschiedene Ämter notwendig macht. Dort erfolgen dann die elektronische Erfassung, Kodierung und Aufbereitung für die Todesursachenstatistik. Dieser Prozess ist zeitaufwendig und fehleranfällig, was zu Verzögerungen und Qualitätsproblemen führt [1]. Auswertungen von Todesbescheinigungen bestätigen, dass die Angaben zur unmittelbaren Todesursache sowie zur Epikrise (Krankheitsverlauf, der zum Tod geführt hat) in vielen Fällen unzureichend sind und z. B. funktionelle Endzustände wie „Herz-Kreislauf-Stillstand“ anstelle einer spezifischen Todesursache eingetragen werden [2–6]. Auswertungen der Todesbescheinigungen von Zweitbegutachtungen bei Feuerbestattungen durch das Universitätsklinikum Hamburg-Eppendorf zeigen, wo bei einem Drittel der ausgefüllten Formulare die Defizite liegen. Fast die Hälfte weist qualitative Mängel (z. B. Angabe falsch oder in falscher Reihenfolge, unzureichende bzw. fehlende Angaben, unklare oder unplausible Angaben) bei der Angabe der Todesursache im ambulanten Bereich auf [2]. Problematisch ist auch die Diagnosestellung auf der Basis eines „Summationstodes“, bei dem mehrere zum Tode führende Krankheiten bzw. Faktoren angegeben werden. Die Diagnoseerstellung stützt sich dabei oft primär auf äußere körperliche Merkmale, sodass ihre inhaltliche Validität aufgrund von fehlenden medizinischen Befunden und Informationen zum Krankheitsverlauf eingeschränkt ist [5].

Zur Verbesserung der Qualität, Vergleichbarkeit und schnellen Verfügbarkeit von Todesursachendaten bieten digitale Lösungen vielfältige Vorteile. Diese umfassen kontextsensitive Menüführung, integrierte Hilfestellungen, automatische Warnungen und Plausibilitätsprüfungen sowie standardisierte und automatisierte Kodierungsprozesse. Die digitale Erfassung von Todesursachen durch Ärztinnen und Ärzte – sowohl ambulant als auch stationär – entlang einer bundeseinheitlichen Eingabemaske könnte eine vereinfachte und beschleunigte Weiterverarbeitung ermöglichen. Solche Innovationen könnten die Erfassung von Todesursachen auf mehreren Ebenen optimieren und effizienter gestalten [7–9]. Digitale Lösungen sind auch erforderlich, um Ressourcen zu schonen, die interne Qualität zu steigern und die Empfehlungen der WHO umzusetzen [10]. Letzteres dient der Verbesserung der Vergleichbarkeit von Gesundheitsdaten auf globaler Ebene [11, 12].

Um die Prozesse im Kontext der Ausstellung von Todesbescheinigungen zu verbessern, wurde im Jahr 2019 durch das Statistische Bundesamt und das Bundesinstitut für Arzneimittel und Medizinprodukte (BfArM) das Projekt „Pilotierung einer bundeseinheitlichen elektronischen Todesbescheinigung (eTB-App)“ [13] initiiert.^1^ Die übergeordneten Ziele des Projekts bestanden in der Digitalisierung zur nachhaltigen Ressourcenschonung, einer Qualitätssteigerung durch Prozessoptimierung, einer Harmonisierung aller 16 Bundesländer sowie einer Harmonisierung zur Erfüllung der geltenden Dokumentationsanforderungen der WHO [14, S. 13 ff.]. Dazu gehört die Erweiterung der Datenerhebung um relevante WHO-Indikatoren, wie z. B. „Kausalkette mit 4 Zeilen“ oder „Angaben zu Operationen vor dem Tod“, die internationale Vergleiche ermöglichen [8, 14].

Das Institut für Digitale Allgemeinmedizin der RWTH Aachen wurde mit dem Projekt zur Erfassung der Nutzungserfahrungen einer bundeseinheitlichen elektronischen Todesbescheinigung beauftragt. Zu diesem Zweck wurde eine quantitative Befragung durchgeführt, um Nutzungshäufigkeit, Bewertung der Bedienungsfreundlichkeit und Identifikation von Änderungswünschen mit der eTB-App zu erfassen. Zusätzlich wurden qualitative Interviews durchgeführt, um vertiefende Einblicke in Alltagstauglichkeit, konkrete Anwendungsprobleme und erforderliche Anpassungen für eine langfristige Nutzung zu gewinnen. Unsere Studie untersucht ausschließlich die digitale Erfassung der Todesbescheinigung im Hinblick auf Nutzen und Bedienungsfreundlichkeit. Sie erlaubt jedoch keine Aussagen zur inhaltlichen Validität der festgestellten Todesursachen.

## Methoden

Im Rahmen der empirischen Untersuchung wurde ein Mixed-Methods-Ansatz mit quantitativer Erhebung in der ersten Erhebungsphase und qualitativen Interviews in der zweiten Erhebungsphase eingesetzt.

### Grundgesamtheit und Rekrutierungsprozess

#### Quantitative Online-Erhebung.

Die Zielgruppe der quantitativen Online-Erhebung bestand aus Ärztinnen und Ärzten aus Baden-Württemberg und Sachsen, die sich vertraglich zur Teilnahme an der Pilotierung der eTB-App (01.02.–30.06.2023) verpflichtet hatten und während der Projektlaufzeit Zugriff auf eines der 30 eTB-Anwendungspakete hatten. Das Anwendungspaket umfasst einen Koffer mit Drucker und Tablet, inklusive Mobilvertrag, sowie länderspezifische Briefumschläge für Todesbescheinigungen. Im ambulanten und insbesondere im stationären Bereich konnten mehrere Personen auf ein eTB-Anwendungspaket zugreifen.

Es wurde eine Vollerhebung durchgeführt. Alle 201 Personen, die die eTB-App in der Pilotphase hätten testen können, wurden per E‑Mail kontaktiert. Im Vorfeld der Teilnahme an der Befragung lag eine Einwilligungserklärung vor. Um die Datenschutzvereinbarungen einzuhalten, erfolgten das Anschreiben sowie der Versand von 2 Erinnerungen per E‑Mail in Sachsen durch das jeweilige Gesundheitsamt und in Baden-Württemberg durch das Statistische Bundesamt. Die eTB-Pilotierung in den beiden Bundesländern wurde aus behördlichen Gründen zeitlich versetzt durchgeführt. Demzufolge erfolgte in beiden Regionen die erste Einladung zur Teilnahme an der anonymen Online-Befragung 3 Wochen nach Projektstart, d. h. nach Erhalt des Anwendungspakets (1. Welle: Februar–März 2023 in Sachsen und Mai–Juni 2023 in Baden-Württemberg; 2. Welle: Juni–August 2023 in Baden-Württemberg und Sachsen).

#### Qualitative Erhebung.

Die qualitativen Interviews wurden (August-September 2023) mit Ärztinnen und Ärzten geführt und decken den ambulanten und stationären Einsatzbereich ab. Die Einladung zur qualitativen Befragung erfolgte zum Ende der Pilotierungsphase. Die Rekrutierung der Ärztinnen und Ärzte erfolgte per E‑Mail durch das beteiligte Gesundheitsamt oder Statistische Bundesamt. Erinnerungsschreiben mit der Bitte um Teilnahme wurden ebenfalls durch die Ämter versandt. Interessierte Ärztinnen und Ärzte konnten dann den Kontakt zum Doktoranden aufsuchen und einen Telefoninterviewtermin vereinbaren. Vor den Interviews wurden alle über Studienzweck, Datenverwendung, -verarbeitung, -speicherung und Teilnahmerechte informiert. Für die qualitativen Interviews wurden schriftliche Einverständniserklärungen zur Aufzeichnung, Transkription und Veröffentlichung der Ergebnisse eingeholt.

### Erhebungsverfahren und -instrumente

#### Online-Erhebung.

Zur Erfassung der Bedienungsfreundlichkeit und Nutzungserfahrung wurde in einer anonymen Online-Befragung eine Kombination validierter Erhebungsinstrumente eingesetzt [15–19]. Die Auswahl umfasste:

1. Module aus der Langversion des modularen Evaluationsfragebogens „Components of User Experience“ (meCUE): 6 Fragen des Moduls I zur „Wahrnehmung, aufgabenbezogener Qualität“ (Bedienungsfreundlichkeit, Funktionsgerechtigkeit, Verständlichkeit, Nützlichkeit, Zielerreichung), 3 Fragen des Moduls II zur visuellen Ästhetik, 1 Frage des Moduls V zur Gesamtbeurteilung, alle Fragen wurden auf einer 7‑stufigen Skala mit spezifischem Bezug zur eTB-App gestellt [15];
2. die Kurzversion des „User Experience Questionnaire“ (UEQ-S): Konstrukte zur pragmatischen und hedonistischen Qualität mittels jeweils 4 gegensätzlicher Adjektivpaare bzw. semantischer Differenziale gemessen mit einer 7‑stufigen Skala;
3. die deutsche Version der Kurzskala zur interaktionsbezogenen Technikaffinität (ATI-S): 4 Fragen zur Erfassung der Technikaufgeschlossenheit mit jeweils 6‑stufiger Skala [19];
4. Fragen der Jameda-Studie: 3 Fragen zum Nutzen der Digitalisierung in der Medizin, 2 Fragen zum Nutzen in der Praxisorganisation, 1 Frage zum Nutzen für Patientinnen und Patienten; alle Fragen gemessen mit einer 5‑stufigen Skala [17];
5. offene Fragen: Eingabe von 3 Änderungs- bzw. Anpassungswünschen, allgemeine Abschlussfrage für weitere Anmerkungen zur eTB-App;
6. demografische Angaben zu den Teilnehmenden: durchschnittliche Anzahl ausgefüllter Todesbescheinigungen in Papierform pro Jahr und eTB-App im Projektzeitraum, Einsatzbereich (ambulant, stationär), soziodemografische Angaben (z. B. Geschlecht, Alter).

Hierbei wurden verpflichtend zu beantwortende Fragen (Muss-Fragen) und optionale Fragen (Kann-Fragen) eingesetzt, um Belastung durch die Befragung zu reduzieren. Die anonyme Online-Befragung wurde direkt vor dem jeweiligen Einsatz einem Pretest unterzogen und anschließend mit einer Bearbeitungszeit von 10 min durchgeführt.

#### Leitfadengestützte Telefoninterviews.

Für die qualitative Erhebung wurde ein leitfadengestütztes Telefoninterview konzipiert. Der Leitfaden umfasste 13 Fragen zu Nutzungserfahrungen, Änderungswünschen und intersektoraler Zusammenarbeit mit Behörden und Institutionen. Im Vorfeld wurde der halbstrukturierte Interviewleitfaden mit 2 Probanden auf Verständlichkeit geprüft.

#### Analyse.

Die quantitativen Daten wurden mit der Software Statistical Package for the Social Sciences (SPSS) ausgewertet. Nicht alle Teilnehmenden haben den Fragebogen vollständig ausgefüllt oder alle Fragen beantwortet, sodass die Fallzahl (*n*) je nach Frage variiert. Bei den deskriptiven Auswertungen und der Bildung additiver Indizes wurden ausschließlich gültige Fälle einbezogen. Fälle mit fehlenden Werten wurden von der Analyse ausgeschlossen. Kategorien mit kleinen Fallzahlen wurden zusammengefasst, um den Datenschutz zu gewährleisten.

Alle qualitativen Interviews wurden aufgezeichnet und transkribiert. Die qualitative Datenanalyse erfolgte mit der Software MAXQDA. Die Kodierung wurde teilweise von 2 Mitarbeitenden durchgeführt, um eine konsistente Kategorisierung zu gewährleisten.

## Ergebnisse

### Quantitative Online-Befragung

#### Rücklaufquote und Eckdaten zu teilnehmenden Ärztinnen und Ärzten.

Von den 201 per E‑Mail kontaktierten Ärztinnen und Ärzten beteiligten sich 44 % (89 Personen) an der online durchgeführten Befragung, welche im Durchschnitt 7 min (Standardabweichung: 4 min) in Anspruch nahm. Die teilnehmenden Ärztinnen und Ärzte (je 50 %; *n* = 70) stammten zu 20 % aus dem ambulanten und zu 44 % aus dem stationären Bereich (*n* = 70). Weitere 36 % der Teilnehmenden waren in beiden Bereichen tätig. Im Rahmen der vorliegenden Untersuchung wurde gefragt, wie viele Todesbescheinigungen in Papierform durchschnittlich im Laufe eines Jahres ausgestellt werden. Hierauf antworteten 43 % der Befragten (*n* = 89), dass sie zwischen 11 und 49 Todesbescheinigungen pro Jahr ausstellen, während weitere 24 % ankreuzten 50 und mehr Todesbescheinigungen pro Jahr auszustellen (Tab. 1).

**Tab. 1:** Rücklauf und Basisinformationen der Teilnehmenden. (Quelle: eTB-App-Begleitstudie 2023, Online-Erhebung)

*eTB-Anwendungspakete*	
Stationärer Bereich	17
Ambulanter Bereich	13
*N*	30
*Rücklaufquote n (%)*	
Teilnehmende	89 (44 %)
Keine Rückmeldung	112 (56 %)
eTB-App zugelassene Testpersonen (*N*)	201 (100 %)
*Geschlecht (%) der Teilnehmenden*	
Weiblich	35 (50 %)
Männlich	35 (50 %)
Divers	0 (0 %)
Gültige Antworten (*n*)	70 (100 %)
*Arbeitsbereich*	
Stationär	31 (44 %)
Ambulant	14 (20 %)
Beides	25 (36 %)
Gültige Antworten (*n*)	70 (100 %)
*Alter in Jahren*	
35 Jahre alt oder jünger	23 (33 %)
36–45 Jahre	27 (39 %)
46–55 Jahre	11 (16 %)
56 Jahre alt oder älter	9 (13 %)
Gültige Antworten (*n*)	70 (100 %)
*Praxisjahre*	
Bis zu 5 Jahre	27 (41 %)
6–10 Jahre	12 (18 %)
11–15 Jahre	14 (21 %)
16–20 Jahre	6 (9 %)
Mehr als 20 Jahre	7 (11 %)
Gültige Antworten (*n*)	66 (100 %)

#### Offenheit und Einstellung zu Digitalisierung.

**Abb. 1:**
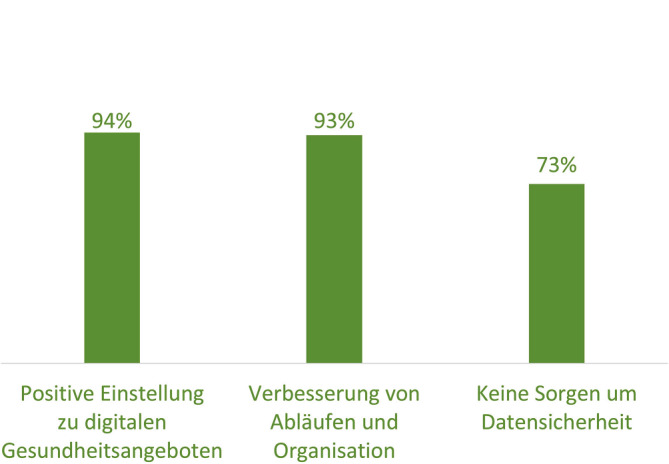
Offenheit und Akzeptanz gegenüber digitalen Angeboten im Gesundheitswesen in %. (Quelle: eTB-App-Begleitstudie 2023, Online-Erhebung; Darstellung der Antwortkategorie „Stimme eher bzw. voll zu“, *n* = 70)

Die Akzeptanz digitaler Gesundheitsangebote war hoch (94 %; *n* = 70), ebenso wie die Zustimmung zur Verbesserung von Abläufen und Organisationsstrukturen (93 %). Die Mehrheit (73 %) hatte keine Sorgen bezüglich der Datensicherheit. Die Gruppen der technikaffinen (53 %) und technikaversen (47 %) Personen waren in etwa gleich groß (Abb. 1).

#### Elektronische Todesbescheinigungen.

**Abb. 2:**
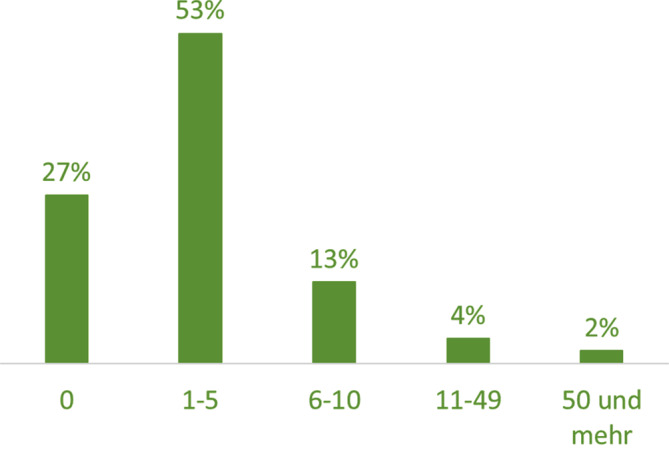
Angaben zur Anzahl der ausgestellten elektronischen Todesbescheinigungen in %. (Quelle: eTB-App-Begleitstudie 2023, Online-Erhebung; Darstellung der Antwortkategorie „Stimme eher bzw. voll zu“, *n* = 89)

Im Befragungszeitraum nutzten 73 % der befragten Ärztinnen und Ärzte (*n* = 89) das bereitgestellte Anwendungspaket für die Ausstellung von elektronischen Todesbescheinigungen. Die Hälfte gab an, 1–5 Todesbescheinigungen digital übermittelt zu haben (Abb. 2).

#### Bedienungsfreundlichkeit und Nutzungserfahrungen.

**Abb. 3:**
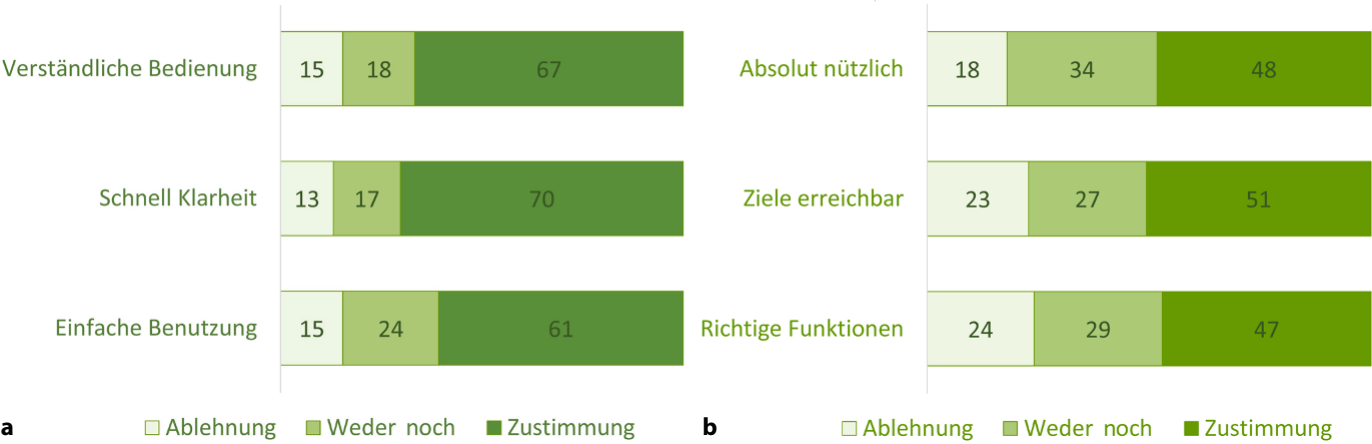
Bewertungen zu Bedienung und Nutzen der eTB-App in %. **a** Bedienung, **b** Nutzen und Funktionsumfang. (Quelle: eTB-App-Begleitstudie 2023, Online-Erhebung; *n* = 84)

Im Zuge der Evaluierung der Nutzungserlebnisse wurde eine Bewertung der Bedienungsfreundlichkeit, Nützlichkeit sowie Attraktivität vorgenommen [15]. Die Ergebnisse der 3 Fragen zur Bedienungsfreundlichkeit der eTB-App sind wie folgt: 67 % der testenden Ärztinnen und Ärzte (*n* = 84) gaben an, dass sie die Bedienung der App verständlich finden, 70 % war schnell klar, wie diese zu bedienen ist, und 61 % fanden die App einfach zu bedienen. Hinsichtlich der Nützlichkeit der eTB-App zeigt sich ein differenziertes Meinungsbild. Etwa die Hälfte der an der Befragung teilnehmenden Ärztinnen und Ärzte bewertet die App als absolut nützlich, sieht ihre Ziele damit erreicht und bestätigt die Verfügbarkeit der als notwendig erachteten Funktionen zur Erstellung einer Todesbescheinigung. Demgegenüber äußern rund 30 % der Befragten eine ambivalente Haltung. Etwa 20 % finden die App nicht bedienungsfreundlich, nicht nützlich und aufgrund fehlender Funktionen nicht zielführend (Abb. 3).

**Abb. 4:**
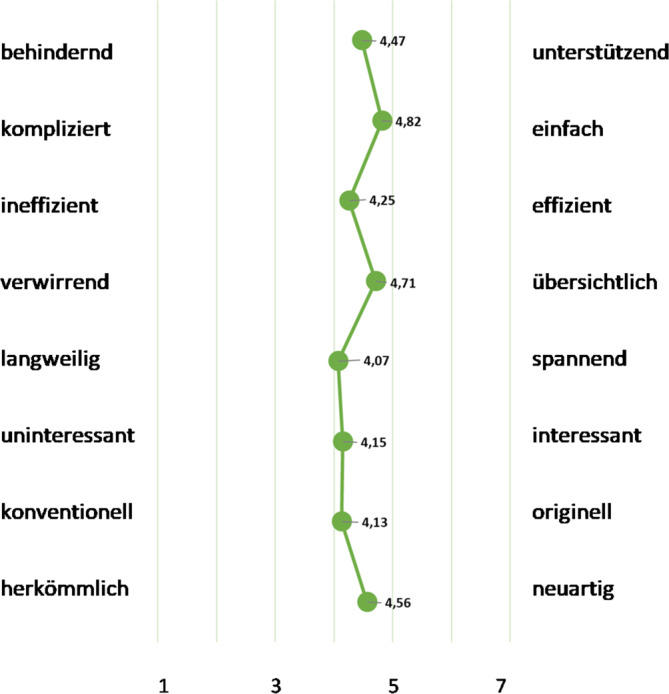
Bewertung der Nutzungserfahrungen anhand des semantischen Differenzials (Mittelwertsangaben) aus dem User Experience Questionnaire (*UEQ*). (Quelle: eTB-App-Begleitstudie 2023, Online-Erhebung; *n* = 55)

Die grafische Darstellung der Ergebnisse des semantischen Differenzials (Abb. 4) zeigt in sämtlichen Aspekten eine klare positive Tendenz, die über dem mittleren Bereich liegt (*n* = 55). Dabei ist hervorzuheben, dass die Ärztinnen und Ärzte, die an der Online-Befragung teilnahmen, die eTB-App mehrheitlich als unterstützend, einfach zu bedienen, übersichtlich und neuartig beschrieben.

Die befragten Ärztinnen und Ärzte konnten Änderungswünsche in ein dafür vorgesehenes Textfeld eintragen. Verbesserungspotenzial in der Anwendung sahen 61 % der Befragten bei der Eingabe der ICD-Codes, 25 % der Befragten würden gerne ganz auf die Erstellung von Papierdokumenten verzichten und weitere 24 % wünschen sich eine vereinfachte Datumseingabe. Weitere Änderungswünsche betreffen die Eingabefelder sowie die Integration in die eigene, vorhandene IT-Infrastruktur. Jeweils 15 % der Befragten wünschen sich eine größere Schrift, um die Eingabe und Lesbarkeit zu verbessern.

### Qualitative leitfadengestützte Interviews

Die qualitativen Daten geben einen ergänzenden und vertiefenden Einblick in die Erfahrungen mit der eTB-App bei Einsatz in der Praxis.

#### Rücklaufquote und Eckdaten zu teilnehmenden Ärztinnen und Ärzten.

An den leitfadengestützten Telefoninterviews nahmen 4 Ärztinnen und 7 Ärzte teil. Die durchschnittliche Gesprächsdauer betrug 20 min (11–35 min). Von den 11 interviewten Personen waren 7 im stationären und 4 im ambulanten Bereich tätig.

#### Bedienungsfreundlichkeit: Technische Aspekte.

Die Aktivierung der eTB-App, beispielsweise die Erstellung eines Accounts, die Registrierung neuer Mitglieder, insbesondere bei häufigem Wechsel im stationären Bereich, die Anfrage nach einem neuen Passwort aufgrund längerer Abwesenheit oder Nichtnutzung, wurde von den testenden Ärztinnen und Ärzten als leicht zu bewerkstelligen beurteilt. Zudem wurde die adäquate bundeslandspezifische Umsetzung der Formulare als intuitiv nutzbar bewertet, was zu einer hohen Zufriedenheit mit dem Programm „eTB-App“ führte. Dies führte zu der Aussage, dass eine technische Einweisung oder Schulung zum Erlernen der Navigation in der App nicht unbedingt notwendig sei. Die Reaktionszeit des technischen Supports auf Anfragen wurde von den Interviewten als unkompliziert, zeitnah und hilfreich bewertet.

„… der durchschnittliche Benutzer mit einer durchschnittlichen technischen Erfahrung …, der wäre sicherlich auch gut ohne diese Schulung zurechtgekommen. … Also das Handout was da mitkam, hat das auch völlig abgedeckt …“ A23CV84 (ambulant).

„Einige meiner Kollegen, wenn sie das Passwort nicht mehr wussten oder wie gesagt halt irgendwas bei der Registrierung nicht funktioniert hatte, bzw. einige Kollegen, die sich neu registrieren lassen wollten, weil wir halt in dem Projektzeitraum auch einige Leute neu eingestellt haben …. Aber das lief wohl immer problemlos.“ K10PV96 (stationär)

#### Qualitätssicherheit bei Datenerfassung – Eingabe und Druck.

Bei der abschließenden Erfassung durch die Ämter sind neben der Lesbarkeit, die Qualitätssicherung sowie unzureichend oder gar nicht ausgefüllte Formularfelder für alle Beteiligten ein Problem. Dies führt zwangsläufig zu einem wiederholten postalischen Umlauf der Dokumente, bis alle Informationen von der behandelnden Ärztin bzw. dem behandelnden Arzt handschriftlich nachgetragen wurden. Bei der Konzeptions- und Entwicklungsphase der eTB-App wurden diese Qualitätssicherheitsmaßnahmen weitestgehend u. a. durch eine Integration von Warnhinweisen, Lexikon, Informations- und Hilfsfelder berücksichtigt. Nahezu alle Ärztinnen und Ärzte thematisieren die automatisierten (Warn‑)Hinweise als große Erleichterung und positives Fortschrittsmoment, z. B.:

„… am Ende vom 12 h Nachtdienst noch den Totenschein mit der Hand ausfüllen, dann schleichen sich einfach Fehler ein. In die falsche Zeile geschrieben. … Da es ja ein amtliches Dokument ist, war es dann meistens so, dass man es dann neu geschrieben hat, … das war schon relativ ärgerlich und zeitaufwendig. … Deswegen war diese Korrekturfunktion sehr praktisch gewesen, dass man nochmal erinnert wird.“ B30SF37 (stationär).

„Dann kann man das Formular ja nicht fertig abschließen, wenn nicht sämtliche Positionen ausgefüllt sind. Das heißt, man kann da nichts vergessen können. Das ist aus Sicht des Standesbeamten Gold wert. Das ergibt keine Rückfragen mehr, ähm Plausibilitäten werden auch ausgemerzt. Man kann es wirklich erst fertigstellen, wenn es richtig komplett und in Ordnung ist.“ O24CH52 (ambulant).

„Weil es so viel Sicherheit gab, wenn da irgendetwas gar nicht passt, dann würde schon das System sozusagen eingreifen.“ K7JF39 (stationär).

#### Probleme bei Kodierungen und Eingabe der Epikrise.

Es zeigten sich einige Hindernisse bei der Nutzung. Zeitaufwendige oder als kompliziert empfundene Eingabefelder veranlassten die Nutzenden teilweise zum Rückgriff auf die Papierform. Insbesondere für ambulant tätige Ärztinnen und Ärzte stellt das Fehlen eines reibungslosen technischen Ablaufs bei der Leichenschau und Ausstellung der Todesbescheinigung vor Ort eine erhebliche Hürde dar. Die Pflichtfelder, die Eingabe der Epikrise in die Freitextfelder sowie die Todesursachenkodierung und Verknüpfung mit ICD-10-Codes wurden in diesem sensiblen Kontext als unnötig umständlich und überarbeitungsbedürftig beschrieben, z. B.:

„Es gibt ja so Codes, wie Hypertonie, die mit der I10. … das gibt man ja relativ oft ein – über die Nebendiagnosen. … da muss man … nicht 10.0 eingeben, weil sonst wird Fehler angezeigt, aber man weiß nicht genau was eigentlich der Fehler ist … Und bei der Niereninsuffizienz ist es irgendwie die N17 und da darf man; muss man aber die Null eingeben, denn sonst wird es auch nicht angenommen. … Das war so ein bisschen tricky da drauf zu kommen.“ A23CV-34 (ambulant).

#### Probleme bei der Datenübertragung zum Drucker.

In der Pilotphase traten Schwierigkeiten bei der Datenübertragung zum Drucker auf, sodass die reibungslose Ausstellung von Todesbescheinigungen sowohl im stationären als auch im ambulanten Bereich nicht immer gewährleistet werden konnte. Insbesondere im Kontext der Ausstellung von Todesbescheinigungen im häuslichen Umfeld wird eine zuverlässige Funktion des Druckers als essenziell erachtet.

„Also mir ist es einmal passiert, das war in einem Plattenbau, … da hat es keine passende Verbindung gegeben zwischen dem Tablet und dem Drucker. Das heißt also, in dem Fall habe ich den Totenschein auf Papier mit der Hand ausgefüllt.“ L5RW39 (ambulant).

#### Implementierung zusätzlicher Schnittstellen.

Um das Potenzial der elektronischen Erfassung von Todesbescheinigungen auszuschöpfen, empfehlen die Ärztinnen und Ärzte die Implementierung weiterer Schnittstellen. Der Zugriff auf die Gesundheitskarte sollte zur Erfassung der Stammdaten und der Epikrise möglich sein. Sie wünschen sich, dass die Vernetzung und Kommunikation nicht nur mit den zuständigen Ämtern (z. B. Standesamt, Gesundheitsamt, Statistisches Landesamt), sondern auch mit weiteren relevanten Stellen wie Bestattungsunternehmen, Versicherungen, Ortspolizei, Justiziariat und Gesundheitsbehörden sichergestellt wird [14, S. 11], z. B.:

„Und ich sage mal ganz perspektivisch, …, die elektronische Gesundheitskarte natürlich für die Authentifizierung oder der elektronische Heilberufe-Ausweis, … auch irgendwo mit genutzt werden könnte.“ K10PV58 (stationär).

„Die Bestatter kamen danach auch alle, das wäre doch cool, wenn wir das auch elektronisch kriegen würden.“ L5IW37 (ambulant).

„Die Polizei …, will mit nicht natürlich(er) oder mit ungeklärt(er Todesursache) … auf jeden Fall ein Dokument. Das könnte … eine Schnittstelle … zwischen diesem Netz und dem … Polizeiserver [sein].“ L5RW123 (ambulant).

#### Gesamtbewertung der Potenziale einer eTB-App.

Die eTB-App überzeugt bereits in der aktuellen Version, die nur eine Schnittstelle zwischen Ärztin bzw. Arzt und einer weiterverarbeitenden Stelle bereitstellt, selbst wenn einige Eingabefelder kritisiert werden. Gleichzeitig wurden Korrektheit und Sicherheit der Dateneingabe, der nachhaltige Verzicht auf Papierdrucke, verkürzte Kommunikationswege und beschleunigte Abläufe bei der Bewertung der App hervorgehoben. Die qualitativen Interviews zeigen, dass es einzelnen Personen schwerfällt, nach der Pilotierungsphase zu manuellen Formularen zurückzukehren. Dies veranschaulicht v. a. das erste Zitat auf eindrückliche Weise:

„Es kotzt mich an, dass ich seit 10 Tagen wieder Totenscheine mit der Hand schreiben muss. Also, ich fand das total cool.“ L5IW15 (ambulant).

„Ich bin, ich bin jetzt 34 Jahre im Beruf. Also Leichenschauen habe ich in meinem Berufsleben so einige gemacht. Und also ich sage mal so von der Benutzerführung, von der Bequemlichkeit der Bedienung ist es, ist es eine gute Sache.“ L5RW31 (ambulant).

„… also für den Anwender Arzt ist es keine, keine Erleichterung im Sinne von einer Zeitersparnis. Unterm Strich ist es die natürlich trotzdem, weil da hintendran natürlich das Standesamt hängt und das Statistisches Bundesamt und die haben es natürlich viel einfacher, wenn das in digitaler Form vorliegt und nicht das Geschmiere der einzelnen ärztlichen Kollegen da entziffert werden muss.“ A23CV68 (ambulant).

„Die Erkenntnis, die ich hatte, dass die Umsetzung der Todesbescheinigung in eine digitale Form wesentlich mehr Potenzial hat, als ich es eigentlich gedacht hätte. … auch Richtung Gesundheitsschutz, Prävention … Also das hat ja, wie gesagt, ein Potenzial. Das hätte ich mir niemals erträumen lassen.“ S26GW-84 (stationär).

## Diskussion

Die Anwendung zur Erstellung einer elektronischen Todesbescheinigung (eTB-App) weist für die teilnehmenden Ärztinnen und Ärzte eine intuitive Menüführung und eine hohe Funktionalität auf. Die Ergebnisse sind bemerkenswert, da sich nur die Hälfte der Befragten als technikaffin klassifiziert (53 %). Nahezu alle online Befragten haben eine positive Einstellung zur Digitalisierung; 94 % befürworten den Ausbau digitaler Gesundheitsangebote und für 93 % kann Digitalisierung zu einer Verbesserung der Patientenversorgung führen. Dieser Wert liegt deutlich über den Ergebnissen der Jameda-Studie (2017), in der nur knapp über die Hälfte der Ärztinnen und Ärzte den digitalen Ausbau befürworteten (53 %) und einen Nutzen für den Praxisalltag (55 %) sahen [17]. In der vorliegenden Studie äußerten lediglich 11 % der Befragten Bedenken hinsichtlich der Datensicherheit. In der Jameda-Studie äußerten mehr als drei Viertel der Ärztinnen und Ärzte, die keine (88 %) oder maximal 2 (75 %) digitale Angebote in ihrer Praxis nutzen, aufgrund von Datenschutzbedenken Nutzungsvorbehalte [17]. Es ist denkbar, dass die Unterschiede stichproben- und zeitabhängig sind. Unsere Befragung erfolgte einige Jahre nach der Jameda-Studie, sodass die App-Testenden inzwischen mehr Vertrauen in digitale Anwendungen gefasst haben könnten.

Eine Analyse der Jahre 2012–2015 zeigt, dass lediglich 2 % der 10.000 untersuchten Todesbescheinigungen keinerlei Fehler aufweisen. In den übrigen 98 % konnten knapp 40.000 Fehler identifiziert werden [20]. Von diesen wiesen 3116 schwere und 35.736 leichte Fehler auf [20]. Bei den schwerwiegenden Fehlern wurden am häufigsten fehlerhafte Kausalketten angegeben (13 %) oder es fehlten sichere Todeszeichen (knapp 3 %; [3, 20]). Problematisch insbesondere im ambulanten Bereich ist die Ausstellung unzureichender Epikrisen mit Eintragungen von Pathomechanismen (z. B. Herzinsuffizienz) oder funktionellen Endzuständen (z. B. „Aetas“ (Alter) oder „Multiorganversagen“ mit der ICD-10-Kodierung R68.8) in der Zeile „Ia“ [5]. Eine fundierte Ausbildung und kontinuierliche Fortbildung sind essenziell für die Verbesserung der Todesfeststellung und -bescheinigung [5]. Dies zeigt sich am Beispiel Bremens, wo bei qualifizierten Ärztinnen und Ärzten eine signifikant höhere inhaltliche Validität der Todesursachenerfassung gefunden wurde [21]. In naher Zukunft könnte in vielen Fällen die elektronische Patientenakte (ePA) genutzt werden, um wichtige Informationen zum Ausfüllen der Epikrise abzurufen und damit die Qualität zu verbessern.

Im Vergleich zur bisherigen Papierversion können mit der eTB-App unleserliche, unzureichende oder falsch ausgefüllte Todesbescheinigungen und damit Rückfragen und Verzögerungen bei der Weiterverarbeitung vermieden werden. Hier helfen Warnhinweise, Plausibilitätstest und kontextsensitive Hilfestellungen beim Ausfüllen. Eine Validierung vor dem Ausdruck, wie die Überprüfung der eingegebenen Daten auf Korrektheit und Vollständigkeit, sorgt für eine höhere Datenqualität. Diese Aspekte wurden von allen Testenden als benutzungsfreundlich und sehr hilfreich bewertet. Die Auswertung von 500 elektronischen Totenscheinen im Vergleich zur Papierform bestätigt die hohe Datenqualität der eTB-App. Zum einen führte die automatische Fehlererkennung zu deutlich plausibleren Daten bei den Standesämtern und Gesundheitsämtern, zum anderen wurde der Anteil der nicht aussagekräftigen Todesursachen bei den eTB-Daten von über 10 % auf 2,8 % deutlich reduziert. Nicht zuletzt wurden „4-zeilige Kausalketten“ und „Angaben zu Operationen“, die zur Weiterleitung an die WHO erforderlich sind, deutlich häufiger angegeben und in über 90 % der Fälle automatisch mit Iris/MUSE kodiert [8].

Digitale Systeme können durch Plausibilitätsprüfung der Kausalkette und Warnhinweise bei Unstimmigkeiten die Qualität der Todesbescheinigung verbessern. Die Validität von Todesbescheinigungen hängt maßgeblich von der ärztlichen Einschätzung während der Leichenschau ab. Durch die elektronische Erfassung werden zwar strukturelle Fehler und Unleserlichkeit minimiert, die medizinische oder juristische Plausibilität der Kausalkette lässt sich jedoch nicht beurteilen. Die Möglichkeit zur nachträglichen inhaltlichen Validierung durch weiterführende Untersuchungen (z. B. Obduktionen) bleibt weiterhin eine Herausforderung [5].

Bei Betrachtung der in Deutschland verwendeten Todesbescheinigungen, welche nicht den Standards der WHO entsprechen und in jedem Bundesland in eigener Ausführung verwendet werden, wird ersichtlich, dass eine valide Erhebung und Vergleichbarkeit von Todesursachenstatistiken nicht nur auf nationaler, sondern auch auf europäischer und internationaler Ebene aktuell nicht gewährleistet werden können. Eine eTB-App verbessert die Qualität durch 3 Faktoren: erstens durch bundesweite Harmonisierung und Standardisierung der Todesbescheinigungen, zweitens durch Reduzierung unzureichend oder nicht ausgefüllter Formularfelder und drittens durch eine einheitliche, an internationale Standards angepasste Kodierung [7].

Im Falle einer Pandemie ist die Verfügbarkeit von Echtzeitdaten für eine optimale Reaktion entscheidend. Länder, die auf digitale Übermittlung von Todesursachenstatistiken setzen, wiesen während der COVID-19-Pandemie im Vergleich zu Ländern mit analoger Übermittlung, wie beispielsweise Deutschland, deutliche Vorteile auf [22]. Denn Todesbescheinigungen sind ein wichtiger Ausgangspunkt für die Gesundheitsbehörden, um zeitnahe Todesursachenstatistiken zu erhalten und Krankheitsausbrüche zu erkennen. Eine kontinuierliche und qualitativ hochwertige Erhebung von Gesundheitsdaten in Echtzeit ist eine Grundvoraussetzung, um Trends zu erkennen und Ressourcen effizienter zuzuweisen [23, 24]. Die Qualität der erhobenen Daten und ihre inhaltliche Validität sind jedoch von entscheidender Bedeutung. Hier kann eine Kombination mit anderen Datenquellen helfen, ein vollständigeres Bild zu erhalten und gezielte Maßnahmen zu ergreifen.

### Limitationen

Nicht alle Teilnehmenden waren im Projektzeitraum im Dienst oder stellten mit der eTB-App Todesbescheinigungen aus. Demgemäß nahmen 27 % der Teilnehmenden ohne eTB-App-Erfahrung an der Befragung teil, da sie Zugang zu einem eTB-App-Anwendungspaket hatten. Dies trägt einerseits zu einer breiteren Perspektive bei, andererseits beeinflusst es auch die Validität einiger spezifischer Nutzungserfahrungen, da die Erfahrungen im Feld fehlen. Schulungen und kollegialer Austausch boten zwar Einblicke in die eTB-App und den Anwendungskoffer, können jedoch die tatsächliche Anwendungspraxis nur bedingt ersetzen. Die Verwendung von Muss- und Kann-Fragen, obwohl methodisch sinnvoll, führte zu Item-Non-Response. Hinsichtlich der Limitationen kann jedoch festgehalten werden, dass die Kenntnis der Größe der Grundgesamtheit eine genaue Berechnung der Rücklaufquote ermöglicht und durch die Vollerhebung die Repräsentativität für die definierte Grundgesamtheit gewährleistet ist. Trotz der hohen beruflichen und zeitlichen Belastung sowie des schwierigen Zugangs zu Ärztinnen und Ärzten konnte eine beachtliche Rücklaufquote von 44 % erreicht werden, was für Befragungen in dieser Berufsgruppe als sehr gut bezeichnet werden kann.

## Fazit

Die leichenschauenden Ärztinnen und Ärzte sowie die beteiligten Gesundheitsämter haben der eTB-App einen hohen Nutzen und Praxistauglichkeit bescheinigt. Ein wesentlicher Vorteil wird in der höheren Genauigkeit und Geschwindigkeit gesehen, mit der die Informationen zur Verfügung stehen. Dies kann Gesundheitsbehörden ermöglichen, Trends früher zu erkennen und potenzielle Gesundheitsprobleme schneller zu identifizieren. Es wurden viele praktikable Verbesserungsvorschläge vorgebracht, wie die Anbindung weiterer Schnittstellen (z. B. zu Beerdigungsinstituten). Weitere Anpassungen sind z. B. bei der Schriftgröße, der Datumseingabe und der hinterlegten ICD-10-Code-Auswahlliste notwendig.

Die Testenden bezeichnen die Rückkehr zur Papierform als Rückschritt. Die im Rahmen der Pilotierung noch erforderliche Papierversion könnte zukünftig durch eine direkte elektronische Übermittlung der Daten an alle Behörden ersetzt werden und somit Zeit und Ressourcen schonen. Eine flächendeckende Implementierung der eTB-App wäre ein richtiger und notwendiger Schritt.

## Footnotes

1 Siehe Video „Wofür braucht Deutschland EINE elektronische Todesbescheinigung?“ unter https://www.bfarm.de/SharedDocs/Videos/DE/etb-pilotierung.html?nn=841312, Zugriff: 19.05.2025.
